# Nepetoidin B from *Salvia plebeia* R. Br. Inhibits Inflammation by Modulating the NF-κB and Nrf2/HO-1 Signaling Pathways in Macrophage Cells

**DOI:** 10.3390/antiox10081208

**Published:** 2021-07-28

**Authors:** Mina Kim, Ji Yeong Kim, Hee Sun Yang, Jeong-Sook Choe, In Guk Hwang

**Affiliations:** Division of Functional Food & Nutrition, Department of Agrofood Resources, National Institute of Agricultural Sciences, Rural Development Administration, Wanju-gun 55365, Korea; lucidminakim@gmail.com (M.K.); 8637914@naver.com (J.Y.K.); yanghs0704@korea.kr (H.S.Y.); swany@korea.kr (J.-S.C.)

**Keywords:** *Salvia plebeia*, nepetoidin B, luteolin, anti-inflammatory effects, ROS, Nrf-2, HO-1, SnPP

## Abstract

*Salvia plebeia* has been used to treat a variety of inflammatory diseases, as well as colds and bronchitis. Macrophages have antioxidant defense mechanisms to cope with the intracellular reactive oxygen species (ROS) produced as part of the immune response. The nuclear factor erythroid 2-related factor 2 (Nrf2)/heme oxygenase (HO)-1 pathway in inflamed macrophages is an appealing target due to its protective effect against ROS-induced cell damage. In this study, nepetoidin B (NeB) was first isolated from *S. plebeia* and identified by nuclear magnetic resonance spectroscopy. NeB reduced pro-inflammatory mediators (nitric oxide and prostaglandin E_2_) and cytokines (tumor necrosis factor-α, interleukin (IL)-6, and IL-1β) in LPS-activated RAW 264.7 cells by inhibiting the NF-κB signaling pathway. In the NeB-treated group, catalase and superoxide dismutase levels were significantly higher, and ROS expression decreased. By activating Nrf2 signaling, NeB enhanced HO-1 expression. Furthermore, when the cells were pretreated with tin protoporphyrin (an HO-1 inhibitor), the anti-inflammatory effects of NeB were reduced. Therefore, NeB may activate the Nrf2/ HO-1 pathway. These results reveal the NeB isolated from *S. plebeia* exerts anti-inflammatory effects by modulating NF-κB signaling and activating the Nrf2/HO-1 pathway in LPS-stimulated RAW 264.7 cells.

## 1. Introduction

Interest in increased life expectancy and quality of life is growing. Homeostasis instability, chronic inflammation, and oxidative stress are linked to the majority of diseases [1]. Inflammation refers to the process by which the innate immune system defends itself against harmful stimuli, including pathogens, toxic compounds, and damaged cells [2]. However, uncontrolled or unresolved inflammation that starts can turn into a long-term problem, contributing to pain and the pathogenesis of various chronic inflammatory disorders [3]. Innate immune cells, particularly macrophages, respond to inflammatory stimulators such as lipopolysaccharide (LPS) [4,5]. LPS initiates a signaling cascade in the nuclear factor-kappa B (NF-κB) pathway and triggers the overproduction of pro-inflammatory mediators and cytokines, as well as reactive oxygen species (ROS) [6]. Pro-inflammatory mediators such as nitric oxide (NO) and prostaglandin E_2_ (PGE_2_) are typical. The activity of enzymes such as inducible NO synthase (iNOS) and cyclooxygenase-2 (COX-2) affects the generation and release of these inflammatory mediators [2]. Furthermore, in macrophages stimulated by LPS, important pro-inflammatory cytokines such as tumor necrosis factor-α (TNF-α), interleukin (IL)-6, and IL-1β are overexpressed, contributing to the pathogenesis of inflammatory disorders [4,6].

Inflammation is also associated with oxidative stress, which increases levels of ROS within cells and modulates enzymes that produce antioxidants [5]. Among several cellular signaling pathways, nuclear factor erythroid 2-related factor 2 (Nrf2) is thought to play a central role in the regulation of stress-related genes, such as heme oxygenase (HO) [5,6]. HO-1 is mediated by Nrf2 and exerts important cytoprotective and anti-inflammatory effects, including inhibition of NO production [7].

With around 980 species, Salvia is the biggest genus in the Lamiaceae family. Members of this genus have been used as herbal medicines worldwide since ancient times [8]. *S. plebeia* is traditionally used to treat inflammatory conditions such as hepatitis, nephritis, bronchitis, and even the common cold [9,10]. A recent study reviewed 93 compounds from *S. plebeia*, including flavonoids, terpenoids, and phenolic acids [10]. Representative active ingredients include 6-methoxyflavones (nepetin, nepitrin, hispidulin, homoplantaginin), luteolin (Lut), and rosmarinic acid [2,9]. In this study, nepetoidin B (NeB), a phenolic compound, was isolated from *S. plebeia* for the first time. Although previous research has identified a variety of biological activities of *S. plebeia* extract, the anti-inflammatory and antioxidant properties of NeB derived from *S. plebeian,* as mediated by mechanisms regulating oxidative stress, remain unknown. Thus, we evaluated the anti-inflammatory effects of NeB isolated from *S. plebeia* in relation to the NF-κB and Nrf2/HO-1 signaling pathway in RAW 264.7 cells. The attenuation of NF-κB has been attributed to the anti-inflammatory activity of Lut, one of the phytochemical constituents of *S. plebeia*; thus, Lut was employed as a positive control against the LPS treatment group [11].

## 2. Materials and Methods

### 2.1. Plant Materials

*S. plebeia* aerial portions were harvested from Buan-gun, Jeollabuk-do, Korea, in January 2020 and authenticated by Dr. In-Guk Hwang of the National Institute of Agricultural Sciences, Rural Development Administration, Korea (voucher specimen: RDA-SP2020-0120).

### 2.2. Reagents and Instruments

All of the solvents used were commercially available high-performance liquid chromatography (HPLC) grade. Analytical grade acetonitrile and water for preparative-HPLC were purchased from Fisher Scientific (Hampton, NH, USA). Formic acid was purchased from Junsei Chemical Co. Ltd. (Tokyo, Japan). Silica gel (70–230 mesh; Merck, Darmstadt, Germany) and Sephadex LH-20 (Sigma-Aldrich, Darmstadt, Germany) were used to perform open column chromatography. YMC J’sphere ODS H-80 (4 µm, 20 × 150 mm; YMC, Kyoto, Japan) was used for the preparative-HPLC. The preparative-HPLC system was conducted on a YMC Forte (YMC, Kyoto, Japan). Proton nuclear magnetic resonance (^1^H-NMR; 600 MHz) and carbon NMR (^13^C-NMR; 150 MHz) spectrometry were performed using the EC-X500 instrument (JEOL, Tokyo, Japan) and CD_3_OD (Cambridge Isotope Laboratories, Tewksbury, MA, USA). Compounds were isolated by ultra-performance liquid chromatography (UPLC; Waters Corp., Milford, MA, USA) and quadrupole time-of-flight mass spectrometry (Q-TOF-MS) with an electrospray ionization source (ESI).

Fetal bovine serum (FBS), Dulbecco’s modified Eagle’s medium (DMEM), and phosphate-buffered saline (PBS) were supplied by Grand Island Biological Company (Grand Island, NY, USA). Penicillin-streptomycin (pen-strep) was purchased from Hyclone Laboratories (Logan, UT, USA). EZ-Cytox was bought from DaeilBio (Seoul, Korea). Griess reagent was supplied by Promega Co. (Madison, WI, USA). BCA protein assay kit, radioimmunoprecipitation assay (RIPA) buffer, protease inhibitor, and phosphatase inhibitor were supplied by Thermo Fisher Scientific (Waltham, MA, USA). Tin protoporphyrin IX chloride (SnPP) was obtained from Cayman Chemical (Ann Arbor, MI, USA). Sigma-Aldrich Co. (St. Louis, MO, USA) provided the following materials: LPS, Lut, paraformaldehyde, Hoechst 33258, and 2′,7′-dichlorodihydrofluorescein diacetate (DCF-DA).

### 2.3. Extraction and Isolation

*S. plebeia* aerial portions were dried (2.7 kg) and extracted using 70% ethanol (27 L) at room temperature over 1 week to obtain a crude extract (1.09 kg). The crude extract was suspended in water before being separated into n-hexane, chloroform, ethyl acetate, n -butanol, and water. In LPS-stimulated RAW 264.7 cells, the inhibitory property of NO was evaluated. The most active ethyl acetate fraction was subjected to column chromatography on silica gel and eluted with chloroform/methanol (100:1→1:1, *v*/*v*) to obtain seven fractions (S1–S7). The S4 fraction was fractionated on Sephadex LH-20 columns with methanol to obtain 10 fractions (L1–L10). The L10 fraction was fractionated via preparative-HPLC, with a gradual increase in methanol (20%→100%), to obtain compound 1 (7.5 mg), which was identified as NeB and used in further experiments. Isolated compound 1 was identified by LC-ESI-QTOF-MS and NMR spectroscopy.

### 2.4. Cell Culture

RAW 264.7 murine macrophage cells (American Type Culture Collection, Manassas, VA, USA) were cultured at 37 °C, in a 5% CO_2_ incubator with DMEM containing 10% FBS and 1% pen-strep solution.

### 2.5. Cell Viability Assay

Cells were seeded into a 96-well plate (4 × 10^4^ cells/well). The cells were treated with various doses of NeB for 24 h. The medium was removed and replaced with fresh medium containing 0.1 μL/mL of EZ-Cytox solution. The cells were further incubated at 37 °C for 2 h. Then, the absorbance was confirmed at 450 nm on a microplate reader (Infinite M200 Pro; Tecan, Krems, Austria). Viability of cells (%) was calculated using the following equation: (absorbance of NeB/mean absorbance of the untreated control) × 100.

### 2.6. Nitric Oxide Assay

Cells were cultured in 96-well plates under the same conditions as previously described. After pretreatment with NeB for 1 h, cells were stimulated with LPS (1 μg/mL) and cultured for 24 h. The cell culture supernatants were collected and mixed with Griess reagent in a ratio of 1:1 and incubated at room temperature for 10 min. Absorbance at 540 nm was confirmed using the Infinite M200 Pro microplate reader. In addition, cells were incubated with or without SnPP (HO inhibitor), and NO levels were evaluated.

### 2.7. Measurement of Cytokines

Cells were activated with LPS (1 μg/mL) for 24 h with or without sample pretreatment with NeB for 1 h. The levels of TNF-α, IL-6, IL-1β, and PGE_2_ were quantified according to the manufacturer’s instructions using enzyme-linked immunosorbent assay kits from R&D Systems Inc. (Minneapolis, MN, USA).

### 2.8. Intracellular Reactive Oxygen Species Generation

DCF-DA dye was used to measure the level of intracellular ROS [12]. Cells were seeded into a black 96-well plate (4 × 10^4^ cells/well) to measure fluorescence intensity or into a 6-well plate (1 × 10^6^ cells/well) for microscopic observation. Cells were pre-incubated with samples for 1 h before LPS stimulation for another 24 h. Next, the cells were added to DCF-DA dye (0.01 mM) and incubated for 10 min. Fluorescence intensity was quantified using the Infinite F200 Pro microplate reader (Tecan) at an excitation wavelength of 510 nm and emission wavelength of 560 nm, and a fluorescence microscope (DMi8; Leica Microsystems, Wetzlar, Germany) was used to visualize the images.

### 2.9. Antioxidant Enzyme Activity

Cells (1 × 10^6^ cells/well) in a 6-well plate were pre-incubated with and without the indicated doses of NeB for 1 h and then stimulated with LPS (1 μg/mL) for 24 h. The medium was removed, and the cells were harvested with lysis buffer (0.1 M Tris-HCl [pH 7.4], 0.5% Triton X-100, 5 mM β-mercaptoethanol, and 0.1 mg/mL phenylmethanesulfonyl fluoride). Then, the cells were centrifuged at 14,000× *g* for 5 min at 4 °C. The supernatants were used for assessing antioxidant enzyme activities. Superoxide dismutase (SOD) and catalase activity were quantified using commercial assay kits from BioVision Inc. (Milpitas, MA, USA), following the manufacturer’s instructions.

### 2.10. NF-κB Binding Activity

Cells were seeded into a 6-well plate (1 × 10^6^ cells/well) and pre-incubated with the samples for 1 h before LPS stimulation for another 1 h. Nuclear extracts were obtained by cell fractionation using a nuclear extraction kit (Thermo Fisher Scientific) for analysis of DNA binding by NF-κB p65. The extracts were further analyzed using an NF-κB p65 transcription factor assay kit (Abcam, Cambridge, UK) in accordance with the manufacturer’s protocol. The activity of NF-κB p65 was evaluated based on the optical density (OD) of the reaction mixture at 450 nm (Infinite M200 Pro).

### 2.11. Immunofluorescence Assay

The nuclear localization of NF-κB was analyzed by immunofluorescence assay. Cells were cultured in 24-well plates (2 × 10^5^ cells/well) on glass coverslips overnight. The cells were treated NeB for 1 h, then stimulated with LPS (1 μg/mL) for another 1 h and fixed in 4% paraformaldehyde for 15 min. After washing, the cells were permeabilized for 10 min in PBS containing 0.5% Triton X-100. BSA (3%, *w*/*v*) prepared in 0.1% Tween 20 was used to keep the cells blocked for 1 h. Then, rabbit anti-mouse NF-κB p65 antibody (1:200, sc-8008; Santa Cruz Biotechnology, Santa Cruz, CA, USA) was incubated for 1 h being reacted with Alexa Fluor 488-conjugated secondary antibody (1:200; sc-8008 AF488; Santa Cruz, CA, USA) for 1 h. After nuclear staining with Hoechst 33258, the coverslips were mounted on the slides. Cells were visualized under the DMi8 fluorescence microscope.

### 2.12. Western Blot Analysis

Cells were solubilized in RIPA buffer containing protease/phosphatase inhibitor (Thermo Fisher Scientific). The protein concentration in the lysate was quantified using a Pierce BCA protein assay kit (Thermo Fisher Scientific). Equal amounts of each protein (20 μg) were separated with 10% sodium-dodecyl sulfate-polyacrylamide gel electrophoresis (Bio-Rad Laboratories Inc., Hercules, CA, USA). Proteins were transblotted onto a polyvinylidene difluoride membrane, which was subsequently blocked by 1 h incubation with 5% skim milk. The membrane was incubated with primary antibodies against iNOS (1:500, sc7271; Santa Cruz, CA, USA), COX-2 (1:500, sc19999; Santa Cruz, CA, USA), NF-κB p65 (1:200, sc8008; Santa Cruz, CA, USA), HO-1 (1:500, sc390991; Santa Cruz, CA, USA), Nrf2 (1:1,000, 12721; Cell Signaling Technology, Danvers, MA, USA), lamin B (1:1000, sc374015; Santa Cruz, CA, USA), and β-actin (1:1000, sc47778; Santa Cruz, CA, USA) for 24 h at 4 °C. Secondary antibodies were diluted to a ratio of 1:5000 and left to bind for 2 h at room temperature. The membranes were treated with enhanced chemiluminescence solution, and bands were visualized using the ChemiDoc system (Bio-Rad Laboratories). Comparative densitometry of the bands was carried out with ImageJ software (NIH, Bethesda, MD, USA) and normalized to the density of the respective actin.

### 2.13. Statistical Analysis

SPSS software (version 20.0; IBM Corp., Armonk, NY, USA) was used to analyze all of the data in this study. Comparisons were performed by analysis of variance (ANOVA) with Duncan’s multiple range tests and independent *t*-tests. A *p*-value < 0.05 was regarded statistically significant.

## 3. Results and Discussion

### 3.1. Isolation of NeB

**Figure 1:**
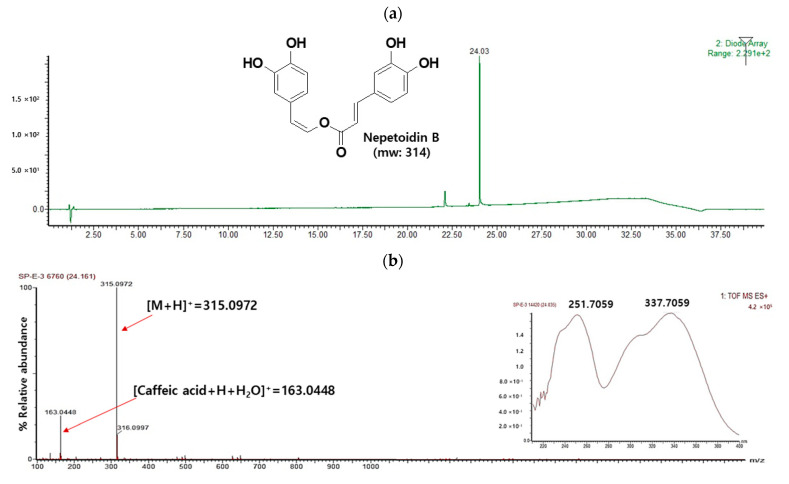
(**a**) UPLC-ESI-TOF/MS chromatogram and (**b**) MS fragmentation of NeB from *S. plebeia*.

During the preliminary screening, the silica gel S4 fraction of the ethyl acetate fraction was a potent inhibitor of NO generation (75% inhibition at 50 µg/mL; data not shown) in LPS-stimulated cells. The S4 fraction was purified repeatedly, and a single compound was successfully isolated (Figure 1a). This compound was structurally characterized in detail by NMR and ESIMS.

The yellow powder of compound **1** was obtained. Its molecular formula was deduced to be C_17_H_14_O_6_ by HRESIMS, with a molecular ion peak [M]^+^ seen at m/z 314.0972 (calcd m/z 314.0790) (Figure 1b). It had a fragment ion at m/z 163, which indicated typical caffeic acid esters [13]. In the ^1^H NMR spectrum, trans-oriented (δ 7.73, 1H, d, J = 16.2 Hz; δ 7.46, 1H, d, J = 16.2 Hz) and cis-oriented (δ 7.23, 1H, d, J = 7.2 Hz; δ 5.63, 1H, d, J = 7.2 Hz) olefinic proton resonances were observed, as well as two sets of protons resonating on the catechol moieties (δ 7.30, 1H, d, J = 2.4 Hz; δ 7.13, 1H, d, J = 1.8 Hz; δ 7.05, 1H, br d, J = 8.4 Hz; δ 6.91, 1H, dd, J = 8.4, 2.4 Hz; δ 6.82, 1H, d, J = 8.4 Hz; δ 6.76, 1H, d, J = 8.4 Hz). In the ^13^C NMR spectrum, 17 carbons were evident: an ester carbonyl carbon at δ 165.9 ppm; 4 oxygenated sp2 carbons at δ 149.1 ppm (C-3), 150.2 ppm (C-4), 146.1 ppm (C-3′), and 146.1 ppm (C-4′); 1 oxygenated sp2 methine carbon at δ 133.0 ppm (C-8′); 2 sp2 qua-ternary carbons at δ 127.9 ppm (C-1), and 127.9(C-1′); and 10 sp2 methine carbons at δ 147.0 ppm (C-8), 133.0 ppm (C-8′), 122.9 ppm (C-6′), 123.6 ppm (C-6), 117.4 ppm (C-2′), 116.7 ppm (C-5), 116.3 ppm (C-5′), 115.7 ppm (C-2), 113.8 ppm (C-7), and 113.3 ppm (C-7′) (Supplementary Figures S1 and S2, Table S1). The ^1^H and ^13^C NMR spectra of compound **1** were compared to those reported in the literature, and compound **1** was identified as NeB. In addition, signals were shown in the UV spectrum at 251 and 337 nm, which is typical for NeB (Figure 1b). NeB has been mainly isolated from *Plectranthus forterim, Salvia miltiorrhiza bunge*, and *Perilla frutescens* [13,14,15]. To the best of our knowledge, NeB has not yet been isolated from *S. plebeia* previously, and this study constitutes the first report of NeB in *S. plebeia*. 

### 3.2. NeB Inhibits the Generation of Pro-Inflammatory Cytokines and Mediators in LPS-Stimulated RAW 264.7 Cells

**Figure 2:**
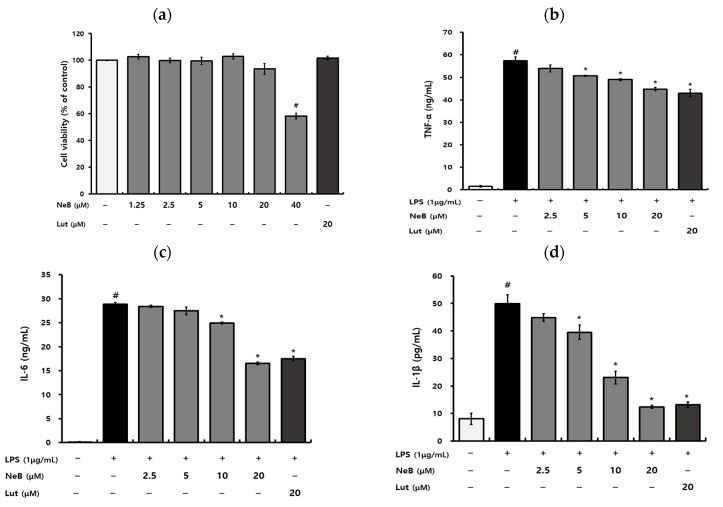
NeB has no effect on cell viability (**a**) but reduces the release of the pro-inflammatory cytokines TNF-α (**b**), IL-6 (**c**), and IL-1β (**d**) in LPS-stimulated RAW 264.7 cells. # *p* < 0.05 compared to the untreated control group; * *p* < 0.05 compared to the LPS-stimulated group. NeB, nepetoidin B; Lut, luteolin (used as a positive control at a concentration of 20 μM).

NeB cytotoxicity on RAW 264.7 cells was assessed via a water-soluble tetrazolium assay using the EZ-Cytox reagent (Figure 2a). The results showed that NeB did not exhibit a toxic effect at concentrations up to 20 μM, but it inhibited cell viability at 40 μM.

The anti-inflammatory potential of NeB was evaluated in the LPS-activated macrophages. Excessive pro-inflammatory cytokines produced by macrophages, such as TNF-α, IL-6, and IL-1β, can cause pathological conditions and are implicated in a variety of chronic inflammatory disorders; hence, these cytokines could be potent biomarkers of the inflammatory process [16]. As shown in Figure 2, pretreatment with NeB decreased the release of TNF-α (Figure 2b), IL-6 (Figure 2c), and IL-1β (Figure 2d) in LPS-activated RAW 264.7 macrophages. In particular, LPS-induced IL-1β was almost entirely suppressed by NeB at 20 μM (Figure 2d).

**Figure 3:**
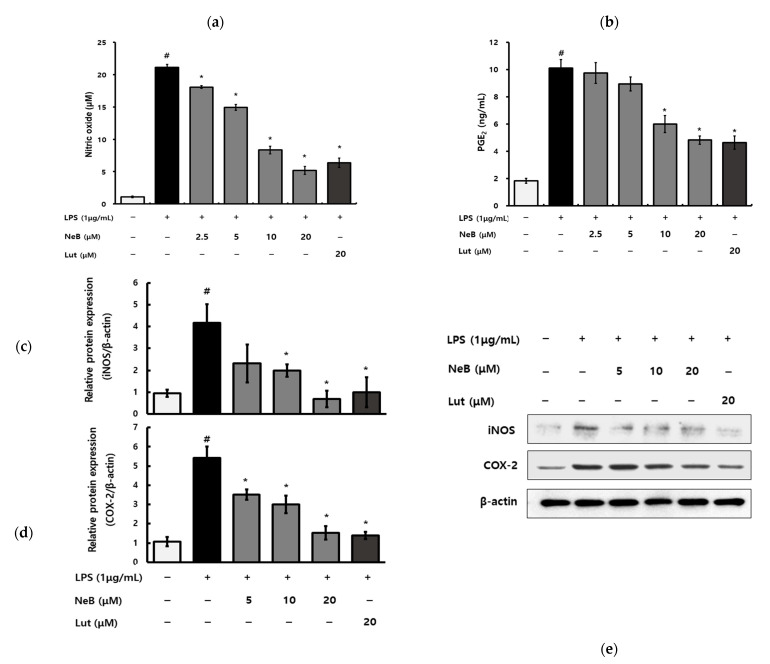
NeB inhibits the generation of NO (**a**) and PGE_2_ (**b**), as well as the protein expression of iNOS (**c**,**e**) and COX-2 (**d**,**e**), in LPS-stimulated RAW 264.7 cells. # *p* < 0.05 compared to the untreated control group; * *p* < 0.05 compared to the LPS-stimulated group. NeB, nepetoidin B; Lut, luteolin (used as a positive control at a concentration of 20 μM).

Moreover, NO and PGE_2_ are well-known pro-inflammatory mediators in many acute and chronic inflammatory disorders [17]. Activated macrophages produce NO as a result of induction by stimuli such as LPS [18]. NO is a key cytostatic and cytotoxic inflammatory mediator and can interact with oxygen-derived radicals to generate toxic components; hence, NO is considered an early indicator of inflammation [19]. PGE_2_ is also an inflammatory mediator, causing the recruitment and activation of inflammatory cells [20]. Therefore, we investigated whether NeB inhibited NO and PGE_2_ production from the RAW 264.7 cells stimulated with LPS. The NO concentration increased considerably followed LPS stimulation, as shown in Figure 3a. Our results show the dose-dependent suppressing effect of NeB on LPS-stimulated NO generation. In macrophages, NeB pretreatment dramatically reduced LPS-induced PGE_2_ generation (Figure 3b). LPS treatment considerably increased the protein levels of iNOS and COX-2, while NeB pretreatment significantly lowered the protein levels of iNOS and COX-2 (Figure 3c–e). iNOS, a key contributor to inflammation via its catalytic effect on NO production, was inhibited by pretreatment with NeB (Figure 3a,c). The regulation of iNOS appears to be involved in NeB’s inhibition of LPS-stimulated NO generation. COX-2 is the primary enzyme controlling PGE_2_ synthesis; the conversion of arachidonic acid to prostaglandins is catalyzed by COX-2 [20]. NeB inhibited COX-2 and PGE_2_ production (Figure 3b,d). The regulation of iNOS and COX-2 was linked to NeB’s inhibitory action on inflammatory mediators such as NO and PGE_2_. Moreover, NeB inhibits both iNOS and COX-2, implying that NF-κB, an activator of both iNOS and COX-2, is involved [18].

### 3.3. NeB Inhibits LPS-Stimulated NF-κB Binding Activity

The transcription factor NF-κB plays a critical role in the development of inflammation; it promotes the expression of inflammatory cytokines [21]. LPS-induced generation of pro-inflammatory mediators involves the NF-κB signaling pathway. In unstimulated cells, members of the NF-κB family bind to inhibitory molecules of the IκB family proteins (NF-κB inhibitors), preventing them from becoming activated [14]. After stimulation, P65 is released from the cytoplasm into the nucleus when IκBα is phosphorylated and then degraded. P65 activates the transcription of inflammatory mediators in the nucleus [21]. Activated NF-κB, which is made up of p50 and p65, is phosphorylated and translocated to the nucleus, where it transcribes inflammation-related genes [19].

**Figure 4:**
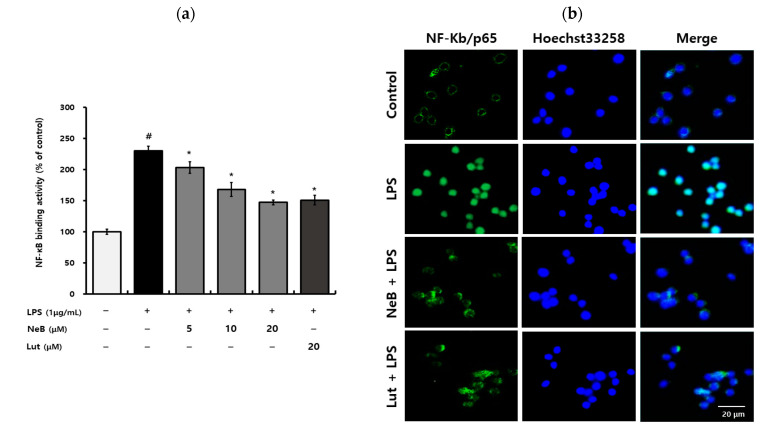
NeB inhibits LPS-stimulated NF-κB binding activity in RAW 264.7 cells. Immunofluorescence assays (**a**) revealed NF-κB DNA binding activity, and immunofluorescence microscopy (**b**) revealed NF-κB nuclear translocation. To access nuclear extracts and confirm NF-κB binding, a commercial NF-κB ELISA kit was employed. After 1 h of NeB (20 μM) treatment, cells were stimulated with LPS (1 μg/mL) for 1 h before being fixed and stained with mouse anti-P65 antibody followed by Alexa-555-labeled anti-rabbit logG (green). DAPI (blue) was used to visualize the nuclei of the relevant cells. Scale bar: 20 µm. # *p* < 0.05 compared to the untreated control group; * *p* < 0.05 compared to the LPS-stimulated group. NeB, nepetoidin B; Lut, luteolin (used as a positive control at a concentration of 20 μM).

Therefore, we aimed to ascertain whether NeB isolated from *S. plebeia* pretreatment affects the activation of the NF-κB signaling pathway in LPS-stimulated RAW 264.7 cells. In this study, NF-κB nuclear translocation was examined using immunofluorescence methods. For 1 h, cells were pretreated with NeB and then activated with LPS. The DNA binding activity of p65 increased the response to LPS stimulation, and pretreatment with NeB attenuated this NF-κB binding activity in a dose-dependent manner in cells (Figure 4a). The results were verified by immunofluorescence microscopy of NF-κB nuclear localization. Figure 4b shows that the p65 subunit was translocated into the nucleus following LPS stimulation, and that pretreatment with NeB blocked this response in RAW 264.7 cells. It was evident that NeB inhibited the inflammatory signaling cascade by regulating the phosphorylation of cytoplasmic p50/p65 units, lowering their ability to translocate into the nucleus and thus limiting the inflammatory signaling cascade. A recent study reported that pretreatment with NeB reduced the NF-κB pathway mainly by affecting nuclear translocation of IKKα/β and NF-κB/p65 [14]. In addition, NeB partially reversed the LPS-induced increases in nuclear p65 and decreased in cytoplasmic IκBα [14].

### 3.4. NeB Inhibits the LPS-Induced Generation of ROS

**Figure 5:**
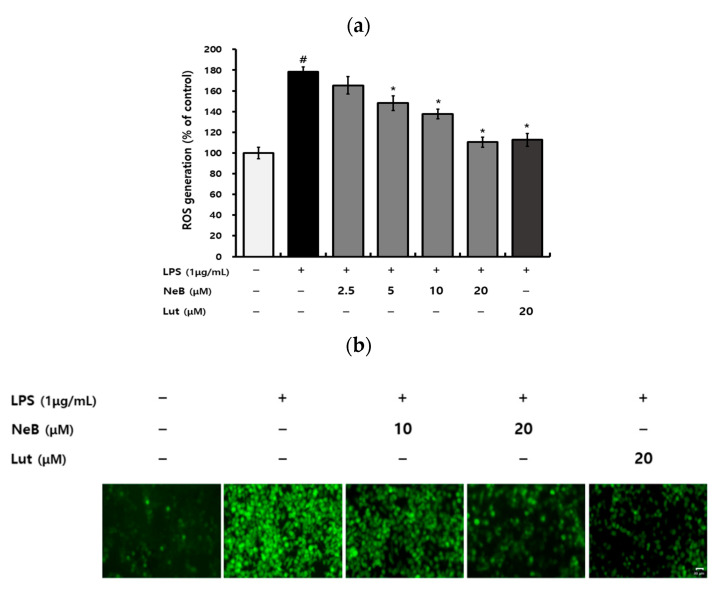

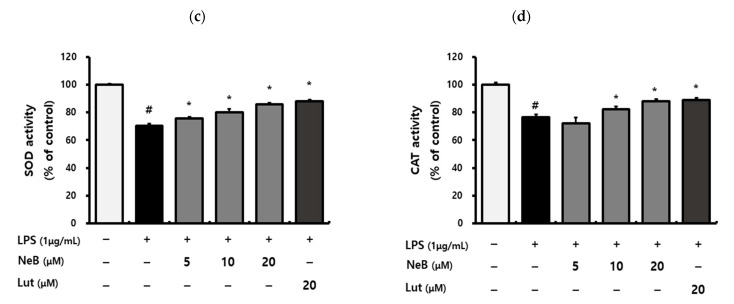
NeB inhibits the production of LPS-stimulated reactive oxygen species (ROS) and stimulates antioxidant enzymes in RAW 264.7 cells. DCF fluorescence (**a**) revealed the level of ROS production, as well as images (**b**) produced by fluorescence microscopy. SOD (**c**) and catalase (**d**) levels of the antioxidant enzymes were determined. Scale bar: 20 µm. # *p* < 0.05 compared to the untreated control group; * *p* < 0.05 compared to the LPS-stimulated group. CAT, catalase; NeB, nepetoidin B; Lut, luteolin (used as a positive control at a concentration of 20 μM).

The ability of *S. plebeia* to down-regulate inflammatory responses has been reported previously [2,22]. However, only a few studies have examined NeB’s anti-inflammatory properties, although Wu et al. (2017) reported that NeB regulated inflammation through the NF-κB pathway. Therefore, this study investigated the potential of NeB isolated from *S. plebeia* to regulate inflammation by modulating oxidative stress alongside its effects on the NF-κB pathway. Chronic inflammation and excessive oxidative stress can cause chronic diseases, such that control of inflammation and oxidative stress is essential [1]. Oxidative stress occurs when the imbalance between the generation of free radicals and their degradation by antioxidants [23,24]. The intracellular ROS levels in macrophages stimulated by LPS increase, initiating oxidative stress [25]. As shown in Figure 5a, LPS significantly increased intracellular ROS levels in cells compared to the untreated control. In contrast, pretreatment with NeB remarkably decreased ROS production in a dose-dependent manner. Furthermore, fluorescence microscopy revealed that LPS increased intracellular ROS levels, and this increase was reversed by pretreatment of the cells with NeB (Figure 5b). These results showed that NeB could reduce ROS accumulation in RAW 264.7 cells.

Because ROS is more chemically reactive than O^2−^^⋅^, it is considered that ROS is the primary cause of cellular damage [24]. O_2_ reduction leads to superoxide O^2−^^⋅^ when electrons from the electron transport chain exit the chain before oxygen are reduced to water at cytochrome *c* oxidase [25]. SOD rapidly transforms O^2−^^⋅^ into H_2_O_2_, which can be degraded and detoxified into H_2_O by the scavenging enzyme catalase [23,26]. Aerobic species have an efficient mechanism for eliminating free-radical species within cells, including vital enzymes such as catalase and SOD [24]. In this study, we evaluated how NeB affects antioxidant enzymes such as SOD and catalase. NeB treatment enhanced the levels of antioxidant enzymes in a dose-dependent manner (Figure 5c,d). NeB previously showed greater free-radical scavenging activity than caffeic acid, rosmarinic acid, and gallic acid [13]. Given that NeB could suppress excessive ROS generation and enhance antioxidant enzymes, the pharmacologic effects of *S. plebeia*-derived NeB could be mediated by their radical scavenging activities.

### 3.5. NeB Attenuates Inflammation by Up-Regulating the Nrf-2/HO-1 Key Signaling Pathway

Aerobic organisms have excellent antioxidant defense networks against oxidative stress, involving primary enzymes such as SOD and catalase, as well as inducible phase II detoxification enzymes such as HO-1, whose expression is regulated by Nrf2 [26]. The antioxidant activity of phenolic compounds is mediated by the cytosolic aryl hydrocarbon receptor (AhR)/Nrf2 pathway [27]. Phenolic compounds bind to the AhR and cause the Kelch-like ECH-associated protein 1 (Keap1)/Nrf2 complex to dissociate, allowing Nrf2 to translocate to the nucleus and promote the transcription of mainstream antioxidant enzymes such as HO-1, SOD, and catalase [28]. Nrf2 activation can effectively reduce inflammation aside from reducing oxidative stress. Nrf2 is a key transcription factor found primarily in the cytoplasm that protects cells from oxidative damage [21]. Nrf2 signaling is considered the most sensitive redox pathway implicated in oxidative injury, and NF-κB is a significant regulator of pro-inflammatory mediators [23]. Redox-sensitive factors Nrf2 and NF-κB are activated by oxidative stress, and a lack of Nrf2 raises oxidative stress and, as a result, promotes cytokine generation [29]. Nutrients related to anti-oxidative stress are reported to induce HO-1 expression [26]. Therefore, it is suggested that HO-1 induction might constitute another mechanism, alongside NF-κB signaling, which may play a role in explaining NeB under oxidative stress conditions concerning the prevention of inflammation.

**Figure 6:**
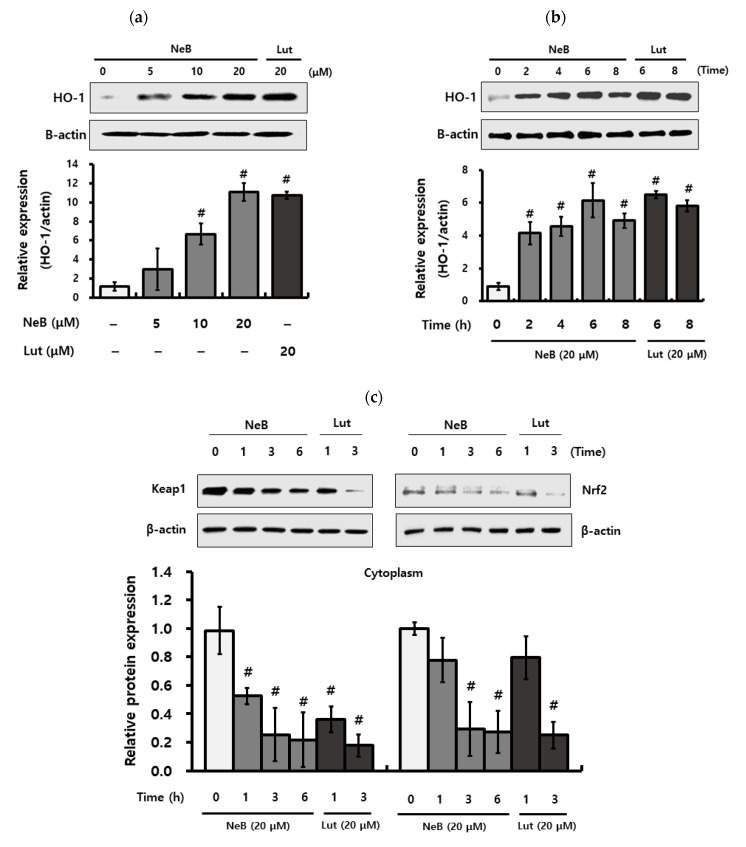

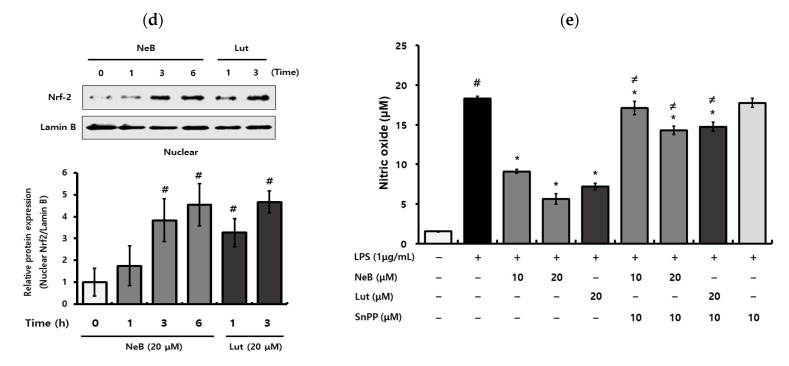
NeB affects inflammation by regulating the Nrf2/HO-1 signaling pathway in RAW 264.7 cells. Western blot analysis was performed to evaluate the expression of HO-1 in a concentration (**a**) and time-dependent (**b**) manner. Protein expression levels of Keap1 and Nrf-2 in the cytosol (**c**) and sequestered Nrf-2 from Keap1 in the nucleus (**d**) were analyzed. The inhibitory effect of NeB on LPS-stimulated NO production related to SnPP (**e**) has been verified. The protein levels were assessed by Western blotting analysis, and the relative levels of protein were quantified by densitometry and normalized to β-actin. The relative expression levels of Nrf2 were quantified by densitometry and normalized to β-actin or lamin B, and the levels of Nrf2 were determined in both the cytosol and nuclei. RAW 264.7 cells were treated with NeB and stimulated with LPS for 24 h after being incubated with SnPP (an HO-1 inhibitor) for 1 h. # *p* < 0.05 compared to the untreated control group; * *p* < 0.05 compared to the LPS-stimulated group; ≠ *p* < 0.05 compared to the sample and LPS-stimulated group. NeB, nepetoidin B; Lut, luteolin (used as a positive control at a concentration of 20 μM).

In this study, NeB significantly increased the protein levels of HO-1 in a dose-dependent manner (Figure 6a). Time-dependent Western blot analyses of RAW 264.7 cells treated with NeB (20 μM) indicated markedly elevated HO-1 protein levels from 2 to 8 h, with a peak at 6 h (Figure 6b).

To further investigate this mechanism, we ascertained the protein levels of Nrf2, a crucial regulator of HO-1 activation. In the cytosol, Nrf2 is sequestered by Keap1 and constitutively targeted for poly-ubiquitination under basal conditions [26]. However, certain stimuli trigger Nrf2 to be released from Keap1, facilitating Nrf2 to translocate into the nucleus and activate HO-1 [30]. Nrf2 is generally coupled with the Keap1 protein and phase II enzyme under normal conditions. HO-1 activation only occurs when Nrf2 is released and translocated to the nucleus [26]. Therefore, we analyzed the protein levels of Nrf2 in the nucleus and cytoplasm to elucidate the mechanism behind NeB-mediated Nrf2 activation. Nrf2 protein levels were influenced by NeB treatment (Figure 6c,d). Under normal conditions, the content of nuclear Nrf2 was significantly increased following NeB treatment, as expected (Figure 6d). In particular, after 6 h, NeB treatment increased Nrf2 nuclear translocation while lowering cytosolic Nrf2 levels. Based on these findings, we inferred that NeB would promote the dissociation of Nrf2 and Keap1, allowing Nrf2 to translocate to the nucleus. Therefore, we suggest that the interaction of NeB with the Nrf2-Keap1 complex may cause HO-1 to be released.

To confirm that NeB inhibits pro-inflammatory mediators such as NO through HO-1, cells were treated with a particular HO-1 inhibitor (SnPP) before NeB treatment. NO production stimulated by LPS was not affected by the addition of SnPP, and inhibition of NO production by NeB was significantly reduced due to the HO-1 inhibitor (Figure 6e). These findings suggest that the antioxidant and anti-inflammatory properties of NeB are associated with activated HO-1, as well as antioxidant enzymes.

In brief, our findings indicated that the anti-inflammatory effects of NeB derived from *S. plebeia* were mediated by the NF-κB and Nrf2/HO signaling pathways in LPS-stimulated RAW 264.7 cells. It was previously reported that Nrf2 and NF-κB are important pathways that regulate the fine balance of cellular redox state and responses to stress and inflammation [31]. Additionally, these two key pathways have functional crosstalk [29]. The absence of Nrf2 can exacerbate NF-κB activity, resulting in increased cytokine generation, whereas NF-κB can regulate Nrf2 transcription and activity, affecting target gene expression in both positive and negative ways [31]. Yerra et al. (2013) suggested that this regulation is perturbed under pathological conditions, which suggests the potential for preventive intervention [29]. Pretreated NeB might modulate crosstalk between two central transcription factors, Nrf2 and NF-κB, and is therefore a potential target for preventing inflammation. Further systemic in vivo studies are required to confirm the influence of NeB and its exact mechanisms of action.

## 4. Conclusions

**Figure 7:**
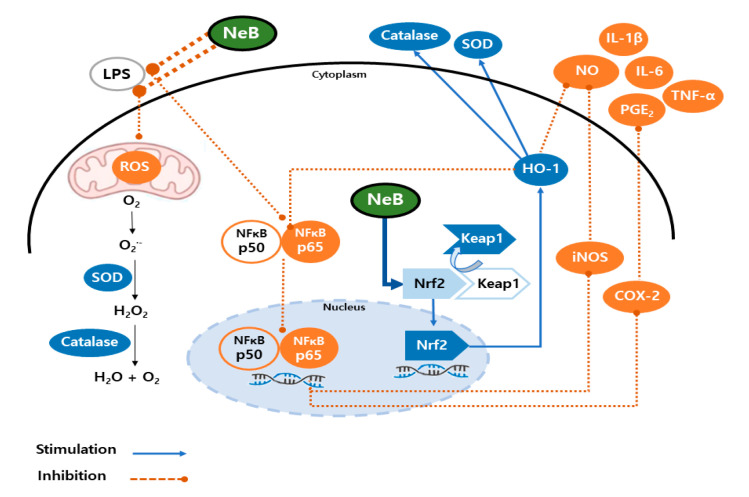
Proposed mechanism for NeB-mediated regulation of inflammation in LPS-stimulated RAW 264.7 macrophage cells. LPS increases ROS generation, which triggers activity in the NF-κB pathway to regulate the levels of iNOS and COX-2. During LPS-stimulated RAW 264.7 macrophage cell activation, pretreatment of NeB reduced the p65 subunit translocation into the nucleus and inhibited NO and PGE_2_ production, along with that of several pro-inflammatory cytokines. NeB also stimulated the nuclear translocation of Nrf2 to induce HO-1 to act as an antioxidant alongside SOD and catalase. NeB, nepetoidin B.

This is the first study to demonstrate the oxidative stress-related anti-inflammatory effects of NeB isolated from *S. plebeia*. Our results show that pretreatment of NeB regulates antioxidant enzymes and inflammatory activity by modulating the Nrf2/HO-1 signaling pathway via inactivation of p38 in RAW 264.7 cells. Given the dual role of Nrf2 activation in preventing oxidative stress and inflammation, activation of the NeB-mediated signaling pathway could modulate crosstalk between two central transcription factors (Nrf2 and NF-κB), which represent a potential preventive target for diseases associated with excessive ROS and pro-inflammatory mediators. The possible signaling pathways of NeB on our results are shown in Figure 7.

## Data Availability

Data is contained within the article and supplementary.

## Supplementary Materials

The following are available online at https://www.mdpi.com/article/10.3390/antiox10081208/s1, Figure S1: ^1^H NMR spectrum of nepetoidin B, Figure S2: ^13^C NMR spectrum of nepetoidin B, Table S1: ^1^H NMR (600 MHz) and ^13^C NMR (150 MHz) data for nepetoidin B.
