# Supplementary material for: Nepetoidin B from Salvia plebeia R. Br. Inhibits Inflammation by Modulating the NF-κB and Nrf2/HO-1 Signaling Pathways in Macrophage Cells
Source: Antioxidants (Basel). 2021 Jul 28;10(8):1208. doi: 10.3390/antiox10081208 (PMC8388923; doi:10.3390/antiox10081208)
Supplement: Supplementary file 1 [file antioxidants-10-01208-s001.zip › antioxidants-1316040-supplementary/nepetoidin NMR (supple. T1).pdf]

Supplementary Table 1. ^{1}H NMR(600 MHz) and ^{13}C NMR (150 MHz) data of nepetoidin B

Position	δ_H (*Int.*, *Multi.*, *J* in Hz)	δ_C
1	- [a]	127.9
2	7.13 (1H, d, 1.8) [b]	115.7
3	-	149.1
4	-	150.2
5	6.82 (1H, d, 8.4)	116.7
6	7.05 (1H, br. d, 8.4)	123.6
7	7.73 (1H, d, 16.2)	113.8
8	7.46 (1H, d, 16.2)	147.0
9	-	165.9
1'	-	127.9
2'	7.30 (1H, d, 2.4)	117.4
3'	-	146.1[a]
4'	-	146.1[a]
5'	6.76 (1H, d, 8.4)	116.3
6'	6.91 (1H, dd, 8.4, 2.4)	122.9
7'	5.63 (1H, d, 7.2)	113.3
8'	7.23(1H, d, 7.2)	133.0

[a] The signals of H-3' and H-4' overlapped.

[b] Proton resonance integral, multiplicity and coupling constants in parenthesis.
