# Supplementary figures and images for: Nepetoidin B from Salvia plebeia R. Br. Inhibits Inflammation by Modulating the NF-κB and Nrf2/HO-1 Signaling Pathways in Macrophage Cells
Source: Antioxidants (Basel). 2021 Jul 28;10(8):1208. doi: 10.3390/antiox10081208 (PMC8388923; doi:10.3390/antiox10081208)

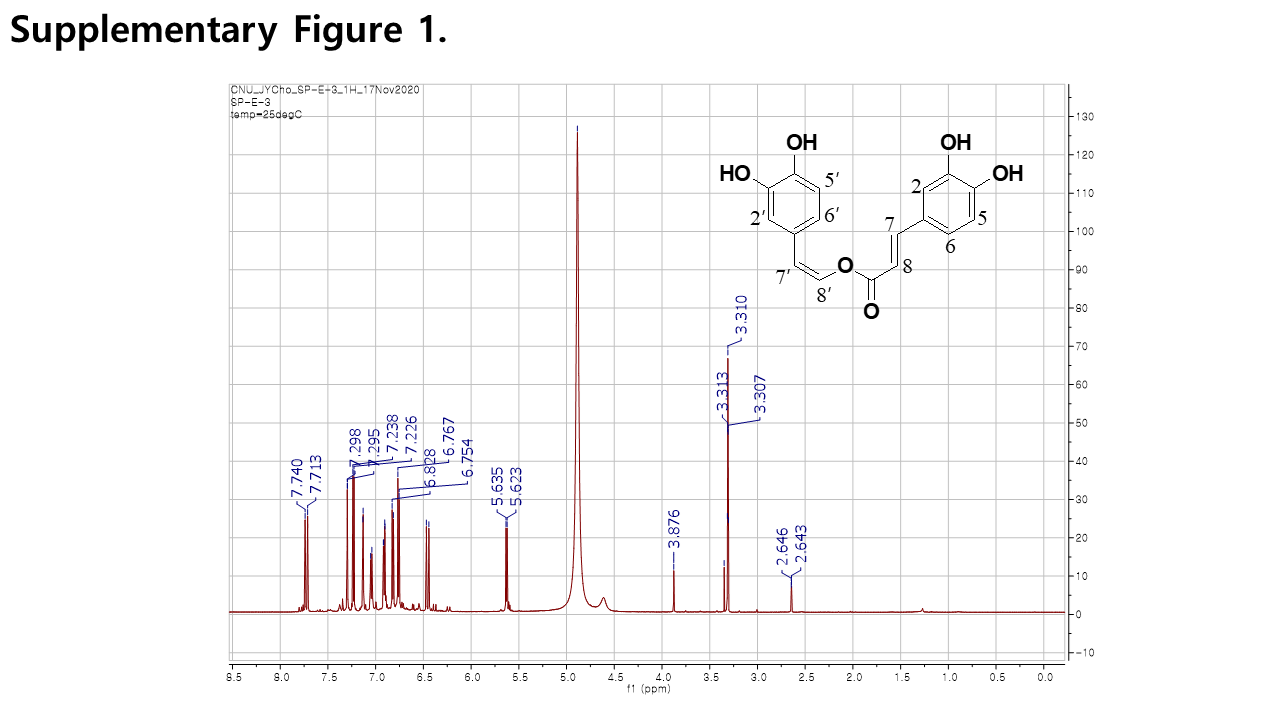

Supplement: Supplementary file 1 [file antioxidants-10-01208-s001.zip › antioxidants-1316040-supplementary/supple. F1.tif]

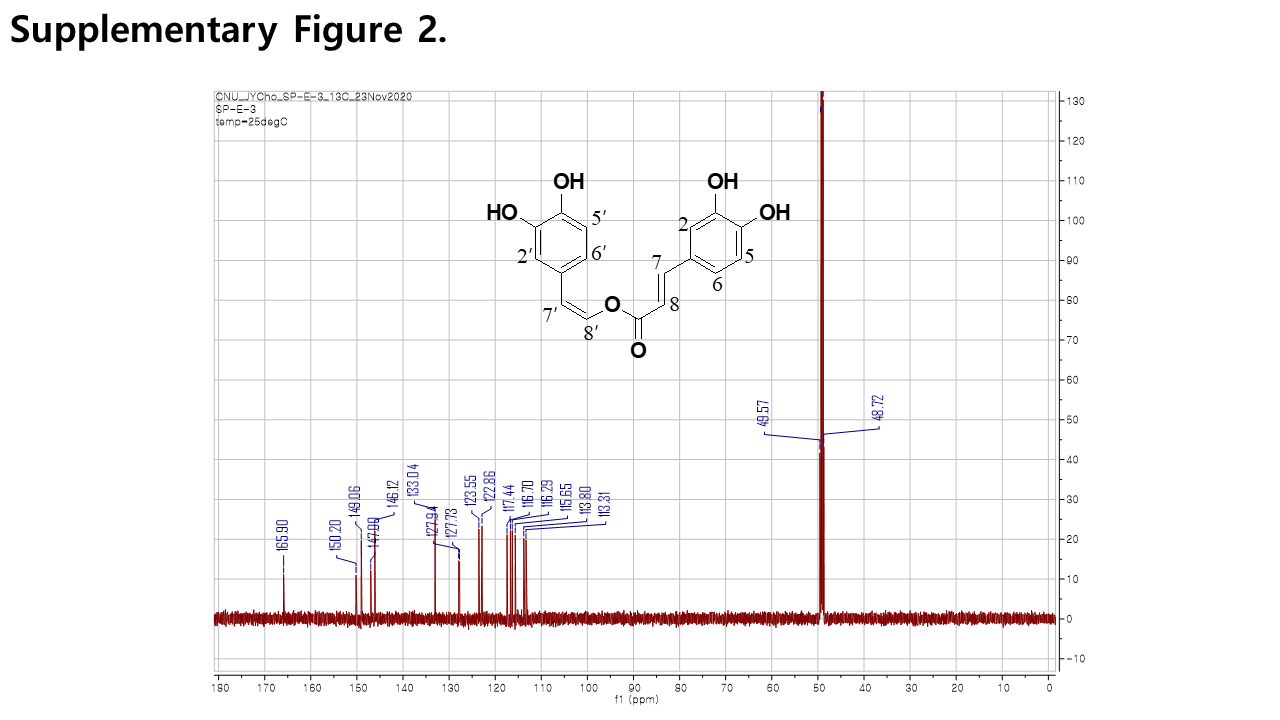

Supplement: Supplementary file 1 [file antioxidants-10-01208-s001.zip › antioxidants-1316040-supplementary/supple. F2.tif]
